# Association between neighborhood deprivation and fruits and vegetables consumption and leisure-time physical activity: a cross-sectional multilevel analysis

**DOI:** 10.1186/1471-2458-13-1103

**Published:** 2013-12-01

**Authors:** Luís Alves, Susana Silva, Milton Severo, Diogo Costa, Maria Fátima Pina, Henrique Barros, Ana Azevedo

**Affiliations:** 1 Institute of Public Health, University of Porto, Rua das Taipas, 135-139, 4050-600 Porto, Portugal; 2 Department of Clinical Epidemiology, Predictive Medicine and Public Health, University of Porto Medical School, Alameda Prof. Hernâni Monteiro, 4200-319 Porto, Portugal; 3 St. André de Canidelo Family Health Unit, Rua das Fábricas, 282, 4400-230 Vila Nova de Gaia, Portugal; 4 Institute of Biomedical Engineering, Rua do Campo Alegre, 823, 4150-180 Porto, Portugal

**Keywords:** Neighborhood deprivation, Socioeconomic position, Fruits and vegetables consumption, Leisure-time physical activity

## Abstract

**Background:**

Most studies of the association between neighborhood socioeconomic deprivation and individual lifestyles leading to cardiovascular disease focused on a single cardiovascular risk factor. The concomitant assessment of more than one risk factor may provide clues to specific mechanisms linking neighborhood disadvantage to individual lifestyles. We investigated the association of neighborhood deprivation with fruits and vegetables consumption and leisure-time physical activity in adults living in an urban center in Portugal.

**Methods:**

In 1999–2003, we assembled a random sample of 2081 adult residents in the city of Porto. Data on sociodemographic characteristics were collected by trained interviewers using structured questionnaires. Fruits and vegetables consumption was estimated using a validated 82-item semiquantitative food frequency questionnaire covering the previous year and expressed in portions per day. Physical activity was evaluated using a questionnaire exploring leisure-time activities over the previous year and expressed in metabolic equivalents (MET).minute/day. Self-reported address was used to place individuals in neighborhoods. Neighborhoods’ socioeconomic characterization was based on aggregated data at the census block level provided by the 2001 National Census. Latent class analysis models were used to identify three discrete socioeconomic classes of neighborhoods. Random effects models with random intercepts at the neighborhood level were used to explore clustering and contextual effects of neighborhood deprivation on each of the outcomes.

**Results:**

We found evidence of neighborhood clustering of fruits and vegetables consumption and leisure-time physical activity that persisted after adjustment for neighborhood deprivation only among women. Women living in the most deprived neighborhoods presented a consumption increase of 0.43 (95% CI: -0.033 to 0.89) portions of fruits and vegetables per day and a decrease in leisure-time physical activity of 47.8 (95% CI: -91.8 to 1.41) MET.minute/day, when compared to those living in the most affluent neighborhoods. Among men, no contextual neighborhood deprivation effects were observed.

**Conclusion:**

Overall, neighborhood deprivation had a small effect on the consumption of fruits and vegetables and leisure-time physical activity. Neighborhood factors other than socioeconomic deprivation may still impact on the studied outcomes among women. This study provides relevant information for the design of interventions directed to neighborhood characteristics in the prevention of cardiovascular diseases.

## Background

In high income countries, an increased incidence of cardiovascular disease among individuals of lower socioeconomic position is a common finding [1]. Inequalities are observed at different levels, from the individual to neighborhoods up to cities and countries. At neighborhood level, socioeconomic characteristics are associated with coronary heart disease (CHD) mortality [2] and incidence [3].

Obesity is an established modifiable risk factor for CHD whose prevalence has been consistently rising [4,5]. Excess weight results from a persistent imbalance between energy intake and expenditure. Although genetic factors play an important role in the development of obesity [6], lifestyle changes, including increased energy intake and sedentariness, seem to explain most of the increase in the prevalence of this condition over the past decades [7,8].

Most obesity research and interventions thereafter have focused on risk factors at the individual level with the aim of improving diet and physical activity patterns through lifestyle changes, and surgical or pharmacological interventions [9]. Although this approach has been partially successful in treating individuals, it has largely failed to modify obesity trends at the population level [10]. Interventions aiming at specific population groups could complement the traditional individual-level interventions, by offering preventive strategies acting more upstream in the causal web. This reasoning is supported by the accumulation of evidence linking neighborhood socioeconomic characteristics and weight status [11]. The literature consistently shows that living in socioeconomically deprived neighborhoods independently increases the odds of excess weight [12-15]. Although in conflicting directions, important lifestyles such as fruits and vegetables intake [16,17] and physical activity [18,19] have also been independently associated with neighborhood socioeconomic characteristics.

Although most research has focused on the study of one cardiovascular risk factor at a time, the simultaneous assessment of more than one risk factor may provide additional clues on specific mechanisms linking neighborhood socioeconomic profile and individual behaviors. We analyzed data from the baseline evaluation of a representative cohort of the adult population of Porto, the second largest Portuguese urban center. First, we examined the crude neighborhood-to-neighborhood variation in the fruits and vegetables consumption and leisure-time physical activity. Second, we evaluated to which extent the adjustment for factors that lead people to reside in a particular neighborhood, for plausible individual-level confounders and neighborhood deprivation changed the between-neighborhood variance in each outcome. Third, we quantified the contextual effect of neighborhood deprivation on each outcome.

## Methods

### Study design and sample selection

The study design has been previously described in detail [20]. Between 1999 and 2003, we assembled a representative sample of community dwellers of Porto, an urban center in the northwest of Portugal with approximately 300,000 inhabitants at that time. Households were selected by random digit dialing of landline telephones. Within each household, a permanent resident aged 18 years or more was selected by simple random sampling and refusals were not replaced. The proportion of participation was 70% [21] and the final sample size was 2485 individuals. The ethics committee of Hospital de São João approved the study. Participants provided written informed consent.

Subjects with missing information on education, marital status, smoking, alcohol intake, abdominal obesity, fruits or vegetables consumption, leisure-time physical activity, or neighborhood socioeconomic class were excluded (n = 404). Thus, the final sample comprised 2081 participants, 1294 women and 787 men. Compared to participants, excluded subjects were younger (mean age: 51.2 vs. 53.2 years, p_Student’s t test_ = 0.01), more frequently sedentary (77.4% vs. 65.2%, p_chi-square test_ < 0.001) and smokers (32.8% vs. 23.0%, p_chi-square_ < 0.001) and less frequently reported excessive alcohol intake (25.6% vs. 34.1%, p_chi-square test_ = 0.02). There were no significant differences regarding gender, education, marital status and daily fruits and vegetables consumption.

### Data collection and definition of variables

Trained interviewers collected data on sociodemographic and behavioral characteristics, using structured questionnaires.

Education was recorded as completed years of schooling and classified in 3 categories: less than 5, 5–11, and more than 11 years (reference). Marital status was grouped as married/living together (reference) and single/divorced/separated/widowed.

Participants were classified as current smokers, including both daily and occasional smokers, or else (reference) [22].

To estimate the lifetime alcohol consumption, participants were asked about the lifetime mean frequency of consumption of different types of alcoholic beverages. The period of highest exposure was considered. The average portion consumed was asked to be lower than, equal to or higher than a glass of 125 ml for wine, a bottle or can of 330 ml for beer, and a cup of 40 ml for spirits. The alcoholic beverages consumption was converted into total alcohol intake assuming the following alcohol concentrations in volume: 12% for wine, 4.7% for beer, 25% for liquors and similar beverages, including Port wine, and 50% for vodka and the like. Two categories of alcohol consumption were defined by the cut points 15.0 grams per day (g/day) for women and 30.0 g/day for men, according to the American Heart Association recommendations [23].

Dietary intake was estimated using an 82-item semiquantitative food frequency questionnaire, covering the previous year. Each participant was asked about the average frequency of consumption (nine categories ranging from “never or less than once a month” to “≥6 times a day”), the average portion consumed (lower than, equal to or higher than the mean portion size) and the seasonal variation of consumption. The reported average portion consumed was based on a photograph manual shown to each participant in which three portion sizes were displayed for each food item. The questionnaire had 16 items related to vegetables and 16 items related to fruits. Only fresh fruits and natural fruit juices, and fresh vegetables and vegetable soups were considered. The food frequency questionnaire had been previously validated by comparison with four 7-day food records, each in a different season of the year [24]. The estimated portions of fruits and vegetables consumed per day were dichotomized using the cut-off of 5 portions per day [25].

Physical activity was evaluated using a questionnaire exploring all professional, domestic and leisure time activities over the past 12 months [26]. Subjects reported the average time per day, week or month spent at rest (sleeping or sitting/lying awake), transport to or from work (walking, motorized vehicle or other), professional activity (very light, light, moderate or heavy) and leisure-time physical activities like watching TV, playing cards or reading (very light activities) as well as exercise (light, moderate or heavy). For the present paper, each group of leisure-time physical activities was assigned a metabolic equivalent (MET) value. One MET corresponded to a resting oxygen consumption of 3.5 ml/kg/min. An average of 1.5, 2.5, 5.0 and 7.0 METs was attributed to very light, light, moderate and heavy activities, respectively [27]. Energy expenditure was estimated by multiplying the related MET value by the self-reported duration of each activity, converted to minutes per day. Participants were considered to be sedentary if they were classified in the lowest sex-specific third of daily leisure or exercise energy expenditure. The cut-off values were 210 and 270 METs.min/day for women and men, respectively.

### Neighborhood socioeconomic assessment

A thorough description of the methods used to characterize neighborhoods from the socioeconomic point of view is presented in the Additional file 1. Briefly, the socioeconomic characterization of neighborhoods in Porto was based on aggregated data at the census block level provided by the 2001 National Census. The census block, broadly equivalent to a city block in an urban setting, constituted the operational definition of neighborhood. For the current analyses, a total of 1662 neighborhoods was considered.

Using different statistical criteria and consensus between three investigators (MFP, DC and LA), we selected 11 variables to characterize neighborhoods regarding age and education/occupation distribution of its residents, and housing characteristics.

Using latent class analysis models, we identified three discrete classes of neighborhoods homogeneous regarding the socioeconomic characteristics. The number of classes was defined according to the Bayesian information criterion, the Akaike information criterion, entropy and interpretability. Starting from one single class and increasing one class at each step, the best solution was identified when the increase in the number of classes did not result in an overall improvement of fit and interpretability. In Table 1, we summarize the socioeconomic characterization of each neighborhood class. The interpretation of the mean values of these variables allowed us to consider neighborhoods classified in the latent classes 1 and 3 as least deprived and most deprived, respectively.

**Table 1:** Characterization of neighborhood latent classes

	**Class 1**		**Class 2**		**Class 3**	
	**n = 386 (23.2%)**		**n = 781 (47.0%)**		**n = 495 (29.8%)**	
	**Mean**	**SE**	**Mean**	**SE**	**Mean**	**SE**
Proportion of retired individuals	16.01	(0.98)	31.26	(0.63)	32.10	(0.82)
Proportion of families with an individual aged ≤15 years	29.53	(1.18)	17.36	(0.45)	27.22	(0.95)
Aging index	111.64	(12.06)	318.41	(14.38)	179.01	(16.79)
Proportion of illiterate subjects	0.98	(0.10)	4.34	(0.29)	9.37	(0.37)
Proportion of subjects with higher education	37.51	(1.18)	15.29	(0.96)	1.94	(0.24)
Proportion of subjects with lower occupation	8.22	(0.77)	23.69	(1.13)	55.08	(1.45)
Unemployment rate	5.40	(0.33)	9.57	(0.28)	15.29	(0.54)
Mean expenditure on housing (owner occupied housing)	403.27	(11.09)	273.50	(8.73)	79.72	(9.56)
Mean expenditure on housing (rented housing)	245.46	(11.38)	109.29	(5.57)	45.77	(2.72)
Attractiveness	25.84	(1.24)	14.62	(0.47)	7.59	(0.60)
Proportion of buildings with reparation needs	32.73	(2.20)	61.71	(1.29)	67.52	(1.66)
	Average latent class probabilities					
Class 1	0.957		0.023		0.000	
Class 2	0.043		0.956		0.043	
Class 3	0.000		0.021		0.957	

SE, standard error.

### Georeferencing procedure

Data on self-reported address was used to place individuals in a specific neighborhood. Georeferencing was made using the streets network, with information on initial and final numbering for each street segment. The position of door entrances was estimated by interpolation on each street segment.

### Statistical analysis

A 2-level hierarchical data structure was considered. In this structure, individuals were nested in neighborhoods. As proposed by Oakes [28], we started by assessing neighborhood-to-neighborhood differences in the health outcomes. This was accomplished by fitting fully unconditional random effects models with random intercepts at the neighborhood level for each of the outcomes of interest (Model 1). These models allowed us to estimate the intraclass cluster coefficient (ICC) that can be interpreted as the proportion of total variance in each outcome that could be attributed to neighborhood factors. Next, we focused on the identification of background factors leading people to reside in a particular neighborhood. As such, linear and quadratic terms for individual age and education, and marital status as a dichotomous variable were added to the models (Model 2). The following step aimed to control for plausible known individual-level confounders for each outcome. As appropriate to each of the studied outcomes, dichotomous variables representing fruits and vegetables consumption, alcohol intake, physical activity and smoking status were also included in the models (Model 3). Finally, we added the variable neighborhood socioeconomic class (Model 4). This modeling sequence allowed us to assess the ICC variation across all models. Furthermore, we were able to estimate the proportion of neighborhood variance that could be explained by the addition of each set of variables using the Model 1 neighborhood variance estimates as reference. Finally, we estimated the neighborhood contextual effect on each outcome by interpreting the beta-coefficients of the neighborhood socioeconomic class variable. The outcome variables were entered in all models as continuous.

## Results

### General individual characterization

Women were slightly younger (mean age: 52.7 vs. 54.2 years, pStudent’s t-test = 0.032) and less educated (median education: 6 vs. 9 years, pMann-Whitney’s test < 0.001) than men. The mean intake of fruits and vegetables was higher in women than in men (mean: 5.6 vs. 5.2 portions per day, p < 0.001). Leisure-time physical activity levels were lower in women than in men, (mean: 376.7 vs. 448.0 MET.min/day, p < 0.001).

In Table 2, we summarize the sample characteristics across neighborhood socioeconomic classes. For both genders, we observed a clear gradient in education across the neighborhood socioeconomic classes. With increasing levels of neighborhood deprivation, we observed both a graded significant increase in the proportion of subjects with the lowest levels of education and a decrease in the proportion of subjects with the highest levels of education. Among women, smoking was more common in less deprived neighborhoods. Among men, excessive alcohol intake was more frequent in more disadvantaged neighborhoods.

**Table 2:** Characteristics of the 2081 participants included in the analyses, by gender and neighborhood socioeconomic class

	**Women**			**Men**		
	Class 1^*^	Class 2^*^	Class 3^*^	Class 1^*^	Class 2^*^	Class 3^*^
	**n (%)**	**n (%)**	**n (%)**	**n (%)**	**n (%)**	**n (%)**
Age						
18-34 years	53 (16.4)	83 (11.0)	25 (11.5)	24 (12.4)	47 (10.7)	21 (13.7)
35-54 years	152 (47.1)	281 (37.3)	101 (46.3)	92 (47.7)	158 (35.8)	57 (37.2)
55-74 years	106 (32.8)	332 (44.1)	80 (36.7)	67 (34.7)	193 (43.8)	60 (39.2)
75 or more years	12 (3.7)	57 (7.6)	12 (5.5)	10 (5.2)	43 (9.8)	15 (9.8)
p	0.017			0.081		
Education						
4 years or less	76 (23.5)	344 (45.7)	133 (61.0)	32 (16.6)	156 (35.4)	72 (47.1)
5 to 11 years	90 (27.9)	204 (27.1)	49 (22.5)	61 (31.6)	164 (37.2)	57 (37.2)
12 or more years	157 (48.6)	205 (27.2)	36 (16.5)	100 (51.8)	121 (27.4)	24 (15.7)
p	<0.001			<0.001		
Marital status						
Married/living together	193 (59.8)	446 (59.2)	142 (65.1)	160 (82.9)	358 (81.2)	125 (81.7)
Else	130 (40.2)	307 (40.8)	76 (34.9)	33 (17.1)	83 (18.8)	28 (18.3)
p	0.273			0.747		
Current smoking						
No	245 (75.8)	654 (86.8)	177 (81.2)	122 (63.2)	306 (69.4)	99 (64.7)
Yes	78 (24.2)	99 (13.2)	41 (18.8)	71 (36.8)	135 (30.6)	54 (35.3)
p		0.025			0.665	
Excessive alcohol intake^†^						
No	265 (82.0)	580 (77.0)	165 (76.7)	108 (56.0)	194 (44.0)	60 (39.2)
Yes	58 (18.0)	173 (23.0)	53 (24.3)	85 (44.0)	247 (56.0)	93 (60.8)
p		0.059			0.001	
Fruits and vegetables consumption						
<5 portions per day	174 (53.9)	410 (54.4)	117 (53.7)	101 (52.3)	211 (47.8)	69 (45.1)
≥5 portions per day	149 (46.1)	343 (45.6)	101 (46.3)	92 (47.7)	230 (52.2)	84 (54.9)
p	0.992			0.173		
Sedentariness^‡^						
No	211 (65.3)	513 (68.1)	130 (59.6)	118 (61.1)	290 (65.8)	92 (60.1)
Yes	112 (34.7)	240 (31.9)	88 (40.4)	75 (38.9)	151 (34.2)	61 (39.9)
p		0.285			0.948	

^*^Neighborhood classes 1 and 3 are interpreted as least deprived and most deprived, respectively.

^†^Alcohol intake >15 g/day for women and >30 g/day for men.

^‡^Leisure-time physical activity ≤210 METs.min/day for women and ≤270 METs.min/day for men.

The results on the association of neighborhood deprivation with fruits and vegetables consumption and leisure-time physical activity are summarized in Table 3 (clustering effects) and Table 4 (contextual effects).

**Table 3:** Neighborhood clustering effects of fruits and vegetables consumption and leisure-time physical activity

	Model 1^*^	Model 2^*^	Model 3^*^	Model 4^*^
**Fruits and vegetables consumption**				
**Women**				
Variance (SE)	0.42 (0.17)	0.39 (0.17)	0.38 (0.16)	0.35 (0.16)
Proportion of explained variance (%)^†^	Reference	7.1	9.5	16.7
ICC (%)	7.0	6.7	6.6	6.1
**Men**				
Variance (SE)	0.22 (0.22)	0.22 (0.22)	0.18 (0.22)	0.18 (0.22)
Proportion of explained variance (%)^†^	Reference	0	18.2	18.2
ICC (%)	4.4	4.4	3.6	3.6
**Leisure-time physical activity**				
**Women**				
Variance (SE)	7521.8 (2013.9)	6328.1 (1706.8)	6203.5 (1695.5)	6333.0 (1677.6)
Proportion of explained variance (%)^†^	Reference	15.9	17.5	15.8
ICC (%)	10.6	10.2	10.1	10.3
**Men**				
Variance (SE)	5310.5 (3721.6)	4033.9 (3399.5)	4135.2 (3391.3)	3631.8 (3343.1)
Proportion of explained variance (%)^†^	Reference	24.0	22.1	31.6
ICC (%)	5.5	4.8	4.9	4.3

SE, standard error; ICC, intracluster correlation coefficient.

^*^Model 1: Null model; Model 2: Model 1 plus adjustment for age, education and marital status; Model 3: Model 2 plus adjustment smoking, alcohol consumption and leisure-time physical activity (fruits and vegetables models) or fruits and vegetables consumption (leisure-time physical activity models); Model 4: Model 3 plus adjustment for neighborhood socioeconomic class.

^†^Proportion of explained variance (%): corresponds to the proportion of between-neighborhood variance that could be explained by neighborhood selection variables, possible confounders and neighborhood socioeconomic class compared to Model 1. For instance, among women 15.9% of the neighborhood variance was explained by neighborhood selection variables: (7521.8-6328.1)/7521.8x100.

**Table 4:** Contextual effects of neighborhood socioeconomic deprivation on fruits and vegetables consumption and leisure-time physical activity

	Fruits and vegetables consumption^*^		**Leisure-time physical activity**^**†**^	
	**β**	**95% CI**	**β**	**95% CI**
**Women (n = 1294)**				
Neighboorhood socioeconomic class 1^‡^	1			
Neighboorhood socioeconomic class 2^‡^	0.24	−0.11 to 0.58	−9.43	−46.4 to 27.6
Neighboorhood socioeconomic class 3^‡^	0.43	−0.033 to 0.89	−47.8	−97.8 to 1.41
**Men (n = 787)**				
Neighboorhood socioeconomic class 1^‡^	1			
Neighboorhood socioeconomic class 2^‡^	−0.23	−0.62 to 0.17	26.6	−25.6 to 78.9
Neighboorhood socioeconomic class 3^‡^	−0.046	−0.55 to 0.46	−12.4	−78.8 to 54.1

95% CI, 95% confidence interval.

^*^Model adjusted for age, education, marital status, smoking, alcohol consumption and leisure-time physical activity.

^†^Model adjusted for age, education, marital status, smoking, alcohol consumption and fruits and vegetables consumption.

^‡^Neighborhood classes 1 (reference class) and 3 are interpreted as least deprived and most deprived, respectively.

### Fruits and vegetables consumption

Among women, we identified a clear neighborhood clustering of fruits and vegetables consumption, with the ICC varying from 7.0% in the null model (model 1) to 6.1% in the fully adjusted model (model 4). The adjustment for neighborhood socioeconomic class further explained 7.2% of variance attributable to neighborhood factors (16.7% _explained variance in Model 4_ - 9.5% _explained variance in Model 3_). Living in a less affluent neighborhood was associated with a daily increase of 0.43 (95% CI: -0.033 to 0.89) portions of fruits and vegetables per day, when compared to the most affluent neighborhoods.

Among men, we observed no evidence of neighborhood clustering or contextual effects on fruits and vegetables consumption.

### Leisure-time physical activity

Among women, we identified a clear neighborhood clustering of leisure-time physical activity, with the ICC varying from 10.6% in the null model (model 1) to 10.3% in the fully adjusted model (model 4). Living in a less affluent neighborhood was associated with a decrease of 47.8 (95% CI: -91.8 to 1.41) MET.min/day, when compared to the most affluent neighborhoods.

Among men, there was less compelling evidence of neighborhood clustering of leisure-time physical activity and no contextual neighborhood deprivation effect was observed.

## Discussion

This study examined the influence of neighborhood deprivation on the consumption of fruits and vegetables and leisure-time physical activity in a sample of community-dwellers of a Portuguese urban center in the early 2000 s. Neighborhood clustering in fruits and vegetables consumption and leisure-time physical activity was only evident among women. Women living in the most deprived neighborhoods presented a marginally non-significant increase in the daily consumption of fruits and vegetables and decrease in leisure-time physical activity levels.

### Clustering effects

The evidence of stronger neighborhood clustering of fruits and vegetables consumption and leisure-time physical activity for women than for men implies that, at least in our setting, women seem to be more susceptible to the influences of the living environment. The fact that fruits and vegetables consumption and leisure-time physical activity are more homogeneous within neighborhoods among women may be explained by gender differences in the construction of identities and role expectations which stress entrenched inequalities in the domestic responsibilities and sports preferences of women and men. Women tend to be more centered on the domestic sphere of the home and family, where their opportunities to engage in social interactions are larger. Additionally, women are more likely to be responsible for maintaining their family’s health and well-being through groceries management or meal conception and preparation [29]. These nurturing activities are influenced by local dietary beliefs and symbolic values which may help to understand gender differences in neighborhood fruits and vegetables consumption clustering. On the other hand, men are usually engaged in more diverse groups outside the domestic sphere contributing to a larger scattering of their spatial exposure. For instance, the practice of collective sports by men typically involves friends or co-workers and implies the spatial dislocation of at least some elements. On the other hand, women tend to engage in more individual activities that are more dependent on the availability of adequate sports facilities at the vicinity level. This may help explain why neighborhood leisure-time physical activity clustering is smaller among men. It is also important to note that approximately 6% and 10% of the variability in fruits and vegetables consumption and leisure-time physical activity, respectively, among women could still be attributed to unmeasured neighborhood characteristics. Thus, further studies are needed to identify its determinants.

### Contextual effects

Among women, the adjustment for neighborhood socioeconomic class further explained approximately 7% of the neighborhood-related variance in fruits and vegetables consumption. Those living in a less affluent neighborhood presented a marginally non-significant increase of 0.43 (95% CI: -0.033 to 0.89) portions of fruits and vegetables per day, when compared to those living in the most affluent neighborhoods. The fact that the spatial density of small food retailers that usually sell fresh fruits and vegetables is much larger among Porto’s most deprived neighborhoods [30] could contribute to the understanding of why fruits and vegetables consumption is higher in the least deprived neighborhoods. Also, eating away from home has been associated with a lower dietary quality [31]. So, if a larger proportion of away meals take place among people living in neighborhoods of increasing affluence, this could also explain the observed neighborhood effect of fruits and vegetables intake. Finally, although we do not have a direct measure of the spatial distribution of vegetable plots across neighborhood socioeconomic classes, in Porto, subsistence agriculture is more common in the most deprived neighborhoods, thus facilitating fruits and vegetables consumption. Our findings are consistent with those of an urban Dutch study [16] in which it was also concluded that participants living in more deprived areas had more favorable fruit consumption patterns than their counterparts in prosperous areas. However, studies in the UK [32] and in the US [16] have mainly concluded that living in more affluent neighborhoods was associated with an increased consumption of fruits and vegetables. It is, thus, apparent, that the association between neighborhood deprivation and fruits and vegetables consumption strongly depends on local factors.

Although the adjustment for neighborhood socioeconomic class did not importantly change the neighborhood-level variance estimate among women, those living in the least affluent neighborhoods had a small decrease in leisure-time physical activity of 47.8 (95% CI: -91.8 to 1.41) MET.min/day, when compared to those living in the most affluent neighborhoods. Unequal distribution of physical activity resources (e.g., walking trails) in more or less deprived neighborhoods is likely to influence opportunities for exercise [33]. Also, gender differences in sports participation [34] may help understand these findings. Although the gender gap seems to be decreasing, women still prefer more aesthetical and individual activities like swimming or aerobics [35,36], which have to be paid and practiced in appropriate facilities which are more scarce in deprived neighborhoods. Finally, compared to women living in the most deprived neighborhoods, those living in more affluent areas may be more susceptible to societal pressures regarding participation in sports that have a strong emphasis on appearance of thinness. This finding is in agreement with the average body mass index being associated with a higher likelihood of reporting body dissatisfaction in neighborhoods of above average affluence [37].

### Strengths and limitations

The results presented in this paper should be interpreted considering the methodological strengths and limitations of this study. First, the cross-sectional nature of this study limits our ability to address causality. However, our analysis did conform to a causal framework. As proposed by Oakes [28], the sequential adjustment for background factors leading individuals to reside in a particular neighborhood and plausible individual-level confounders is a transparent methodological approach to address compositional neighborhood effects. To the extent that these models are successful in addressing confounding between people in neighborhoods, our fully adjusted model should provide valid estimates of neighborhood contextual effects. Second, we are confident that most of our findings are still relevant in the present, even though the sovereign debt crisis that affected Portugal over the last years may have changed the relation between neighborhood deprivation and individual lifestyles linked to cardiovascular disease. Third, our study was under-powered to detect statistically significant contextual effects. However, the association of area socioeconomic position with each outcome variable was in the expected direction, thus while a larger study may have found statistically significant area effects due to increased precision, it is unlikely that a larger sample would have reached substantially different conclusions. Fourth, our definition of neighborhood was based on artificial administrative territorial divisions for analytical convenience rather than for reasons that were hypothesized to influence each outcome. These facts probably lead to an underestimation of area socioeconomic position effects.

## Conclusion

This study contributes to judge the potential of interventions directed to neighborhood socioeconomic characteristics to prevent cardiovascular diseases. To the extent that we were able to adjust for neighborhood selection factors and confounding factors, we estimated both the neighborhood clustering and the contextual effects of neighborhood deprivation on two important modifiable cardiovascular risk factors. Overall, interventions aimed to decrease neighborhood deprivation are expected to have a small impact on the studied outcomes. Still, women in deprived neighborhoods had higher fruits and vegetables consumption and lower leisure-time physical activity levels. Also, neighborhood factors other than socioeconomic deprivation may still impact on these outcomes among women. However, further studies are necessary to identify which modifiable neighborhood characteristics explain these clustering patterns.

## Competing interests

The authors declare that they have no competing interests.

## Authors’ contributions

All authors have contributed substantially to the conception and design, acquisition of data, or analysis; LA have drafted the article; LA, MFP, DC and MS performed the socioeconomic characterization of neighborhoods; LA, MS and AA performed the statistical analysis for the associations between neighborhood deprivation and each outcome; SS, MS, DC, MFP, HB and AA critically revised the article; All authors have given approval of the final version to be published.

## Pre-publication history

The pre-publication history for this paper can be accessed here:

http://www.biomedcentral.com/1471-2458/13/1103/prepub

## Supplementary Material

Additional file 1 **Neighborhood socioeconomic assessment.** A detailed description of the methods used to characterize neighborhoods from the socioeconomic point of view. Click here for file
