# Supplementary material for: Association between neighborhood deprivation and fruits and vegetables consumption and leisure-time physical activity: a cross-sectional multilevel analysis
Source: BMC Public Health. 2013 Dec 1;13:1103. doi: 10.1186/1471-2458-13-1103 (PMC3879067; doi:10.1186/1471-2458-13-1103)
Supplement: Additional file 1 — Neighborhood socioeconomic assessment. A detailed description of the methods used to characterize neighborhoods from the socioeconomic point of view. [file 1471-2458-13-1103-S1.pdf]

Neighborhood socioeconomic assessment

The socioeconomic characterization of neighborhoods was based on aggregated data provided by the 2001 National Census regarding Porto. Authorization to access to this database was granted from Statistics Portugal. The unit of observation was the census block, which is broadly equivalent to a city block in an urban setting [1]. The area of the city of Porto is 41.7km^2 and this territory has been divided into 2064 census block [2]. We excluded 402 census blocks in which 10 or less individuals lived at the time of the census evaluation. Thus, these analyses were based on 1662 census blocks which constituted the operational definition for neighborhoods.

Each neighborhood was characterized by a set of variables related to buildings, households, families and individuals. Most of these variables were provided as absolute counts for each neighborhood (e.g.: number of women aged 65 years or more, number of persons with 4 complete years of education). To allow for the comparison between neighborhoods, we calculated proportions for the variables to be included in the statistical analysis. For example, to create the indicator "proportion of residents with higher education", we divided the absolute number of residents with university degree or higher by the absolute number of residents. Using this information, we assembled the largest possible group of indicators thought to plausibly represent different socioeconomic aspects of neighborhoods. This initial database comprised a total of 47 indicators. After an initial descriptive analysis, we noted that some indicators had important floor or ceiling effects [3]. By limiting heterogeneity, the asymmetries in the distribution of these indicators limited their contribution to the characterization of socioeconomic inequalities. Therefore, we excluded 15 indicators for which 20% or more of the neighborhoods presented the minimum or maximum possible value. Next, after observing correlations ≥0.8 within three subsets of the 32 remaining variables (subset 1: 4 variables, subset 2: 3 variables and subset 3: 2 variables), we selected only one variable from each of the subsets by consensus between investigators and excluded the remaining ones. This procedure led to the exclusion of 6 variables. After evaluating the remaining dataset, there was enough information to characterize three distinct socioeconomic dimensions: age composition of residents within neighborhoods, education/occupation and housing characteristics. Three to four variables

were chosen for each dimension in order to ensure a minimum reliability of 70% and to avoid overrepresentation of specific socioeconomic dimensions. The selection of these variables was first performed independently by three investigators (FP, DC and LA) and then decided by consensus, resulting in the exclusion of 15 variables. The final dataset comprised the following 11 variables: proportion of retired individuals, proportion of families with a person aged 15 years or less, aging index, illiteracy proportion, proportion of subjects with higher education, proportion of subjects with lower occupation, unemployment rate, mean expenditure on housing (owner occupied housing), mean expenditure on housing (rented housing), attractiveness (proportion of residents that resided in another territorial unit or country 5 years before) and proportion of buildings with reparation needs. The process of variable selection is summarized in Supplementary Figure 1. The operational definition of each of the 11 variables is presented in Supplementary Table 1.

Statistical analyses

In order to characterize neighborhoods, we fitted latent class analysis models to identify discrete classes of neighborhoods homogeneous regarding the socioeconomic characteristics. In this study, the number of latent classes was defined according to the Bayesian information criterion (BIC), the Akaike information criterion (AIC), entropy and interpretability. Better fit was indicated by smaller AIC and BIC values and entropy values closer to 1.0 [4]. Starting from one single class and increasing one class at each step, the best solution was identified when the increase in the number of classes did not result in an overall fit and interpretability improvement. Interpretation of the model is usually based on item profiles in each category, obtained from the probabilities of endorsing each item response conditional on class membership.

Characterization of neighborhood socioeconomic classes

We summarize the socioeconomic characteristics of each neighborhood class in Table 2 (main article). Neighborhood type 1 (class 1) accounted for 23.2% (386/1662) of the total number of analyzed census blocks. These neighborhoods were characterized by the lowest percentage of retired residents and highest percentage of families with young individuals,

which translated into the lowest aging index. They were also characterized by the lowest proportions of illiteracy, unemployment and of residents engaged in low occupations. Conversely, these neighborhoods presented the highest proportion of residents with higher education, the highest levels of attractiveness, housing expenditure and the smallest proportion of buildings with reparation needs. Neighborhood type 2 (class 2) accounted for 47.0% (781/1662) of the total number of analyzed census blocks. These neighborhoods were characterized by a large percentage of retired residents but smallest percentage of families with young individuals, translating in the highest aging index. These neighborhoods were also characterized by intermediate proportions of illiteracy, unemployment, low occupation and higher education. Finally, these neighborhoods had intermediate levels of attractiveness, housing expenditure and a high proportion of buildings with reparation needs. Neighborhood type 3 (class 3) accounted for 29.8% (495/1662) of the total number of analyzed census blocks. These neighborhoods were characterized by a medium aging index, reflecting the mixture of both retired residents and families with young individuals. These neighborhoods presented the worse indicators of illiteracy, higher education, low occupation and unemployment. They also presented the lowest levels of attractiveness, housing expenditure and the largest proportion of buildings with reparation needs.

References

1. **Meta-informação: subsecção estatística** [http://smi.ine.pt/Conceito/Detalhes/1926]

2. INE: *Tipologia socio-económica da Área Metropolitana do Porto - 2001.* Porto: Instituto Nacional de Estatística; 2004.

3. Austin PC, Brunner LJ: **Type I Error Inflation in the Presence of a Ceiling Effect.** *Am Stat* 2003, **57:**97-104.

4. Collins LM, Lanza ST: *Latent class and latent transition analysis : with applications in the social behavioral, and health sciences.* Hoboken, N.J.: Wiley; 2010.

Supplementary Figure 1: Flowchart representing the sequential criteria used to select the variables to characterize neighborhoods

47 variables (largest possible group of variables thought to plausibly represent socioeconomic aspects of neighborhoods)

15 variables excluded due to floor or ceiling effects

32 variables (after observing correlations ≥0.8 within three subsets of the 32 variables (subset 1: 4 variables, subset 2: 3 variables and subset 3: 2 variables), we selected only one variable from each of the subsets by consensus between investigators and excluded the remaining ones)

6 variables excluded with correlations ≥0.8 (three variables from subset 1, two variables from subset 2 and one variable from subset 3)

26 variables (this set of variables was thought to represent three distinct socioeconomic dimensions: age, education/occupation and housing characteristics)

15 variables excluded to ensure a minimum reliability of 70% and to avoid overrepresentation of specific socioeconomic dimensions

11 variables (final dataset)

Supplementary Table 1: Operational definition of the 11 variables selected to socioeconomically characterize neighborhoods

A priori construct	Selected variables	Numerator	Denominator
Age	Proportion of retired individuals	Number of retired persons	Number of residents
	Proportion of families with person aged 15 years or less	Number of families with an individual aged 15 years or less	Number of resident families
	Aging index	Number of persons aged 65 years or more	Number of residents aged 0-14 years
Education or occupation	Illiteracy proportion	Number of persons that cannot read or write	Number of residents aged ≥10 years
	Proportion of subjects with higher education	Number of persons with university degree or higher	Number of residents
	Proportion of subjects with lower occupation	Number of persons with low skilled occupation	Number of residents
	Unemployment rate	Number of unemployed persons	Number of residents aged ≥15 years
Building characteristics	Mean expenditure on housing (owner occupied housing)	Not applicable	
	Mean expenditure on housing (rented housing)	Not applicable	
	Attractiveness	Number of persons that resided in another territorial unit 5 years before	Number of residents
	Proportion of buildings with reparation needs	Number of buildings with reparation needs	Number of buildings
